# Size Specific Transfection to Mammalian Cells by Micropillar Array Electroporation

**DOI:** 10.1038/srep38661

**Published:** 2016-12-07

**Authors:** Yingbo Zu, Shuyan Huang, Yang Lu, Xuan Liu, Shengnian Wang

**Affiliations:** 1 Chemical Engineering, Louisiana Tech University, PO Box 10137, Ruston, LA, 71272, USA; 2 Institute for Micromanufacturing, Louisiana Tech University, PO Box 10137, Ruston, LA, 71272, USA; 3 Biomedical Engineering, Louisiana Tech University, PO Box 10137, Ruston, LA, 71272, USA; 4 Center for Biomedical Engineering and Rehabilitations, Louisiana Tech University, PO Box 10137, Ruston, LA, 71272, USA

## Abstract

Electroporation serves as a promising non-viral gene delivery approach, while its current configuration carries several drawbacks associated with high-voltage electrical pulses and heterogeneous treatment on individual cells. Here we developed a new micropillar array electroporation (MAE) platform to advance the electroporation-based delivery of DNA and RNA probes into mammalian cells. By introducing well-patterned micropillar array texture on the electrode surface, the number of pillars each cell faces varies with its plasma membrane surface area, despite their large population and random locations. In this way, cell size specific electroporation is conveniently carried out, contributing to a 2.5~3 fold increase on plasmid DNA transfection and an additional 10–55% transgene knockdown with siRNA probes, respectively. The delivery efficiency varies with the number and size of micropillars as well as their pattern density. As MAE works like many single cell electroporation are carried out in parallel, the electrophysiology response of individual cells is representative, which has potentials to facilitate the tedious, cell-specific protocol screening process in current bulk electroporation (i.e., electroporation to a large population of cells). Its success might promote the wide adoption of electroporation as a safe and effective non-viral gene delivery approach needed in many biological research and clinical treatments.

Gene induction and/or inhibition provide powerful tools to understand gene functions^1^, control cellular signals^2^, and develop new therapeutic technologies^3^. The emerging exploration in RNA interference^4^,^5^ and cell reprogramming^6^,^7^ for cancer treatment and/or personalized medicine pushes the expectation on the effectiveness of gene delivery to a new high level. Safe delivery of healthy copies of DNA or RNA probes in majority treated cells with high efficiency and excellent survival rate becomes essential for the success of these applications. Viral transduction is highly stable and efficienct^8^, but has limited carrying capacity and high risk of oncogenesis and inflammation^9^. This largely stimulates the pursuit of nonviral delivery strategies, including both chemical and physical approaches, which however have not yet become competitive to their viral counterpart^10^,^11^,^12^,^13^,^14^. Compared to the chemical delivery strategies, physical approaches grew fast in recent years, benefited from their direct delivery to desired intracellular locations^15^,^16^,^17^,^18^,^19^. Among them, electroporation is often favorable for its balance of simplicity, transfection effectiveness, broad allowance on probe or cell types, and operation convenience^20^,^21^,^22^. In electroporation, short, high-voltage electric pulses are applied to surpass the cell membrane capacitance, making the subjected cells transiently permeable^20^. It has two active but relatively independent research directions: single cell electroporation (SCE) and bulk electroporation (BE). The former focuses on the discovery of cellular transport dynamics and mechanism (i.e., electrophysiology) while the latter targets at high transfection efficiency to cells in a large population. Both fields are important but difficult to support each other. For example, according to single cell electroporation theory, the transmembrane potential (Δ*V*_*m*_, in V) for reversible breakdown of the cell lipid bilayer can be estimated by:


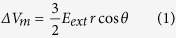


where *E*_*ext*_ is the electric field strength (in V/cm), *r* is the radius of cell (in cm), *θ* is the angle between *E*_*ext*_ and the membrane surface. For a 10-μm cell, a pulse of ~267 V/cm (i.e. ~54 V across electrodes separated by 2 mm) is enough for successful cell permeabilization. However, the practical pulse strength adopted in most bulk electroporation protocols is 0.5~1.0 kV/cm for mammalian cells and varies with cell type, source, and population^20^,^21^,^22^. The available protocols are established by trial-and-error, instead of equation (1), at a compromise of acceptable transfection efficiency and cell viability. The high-voltage pulses, though effective in improving the cell membrane permeability and probe uptake, inevitably leads to severe side effects detrimental to later cell survival^23^,^24^,^25^.

A number of new electroporation setups with micro-/nanoscale features have recently been introduced to tackle these issues, either through closely patterning electrode pairs (e.g. ~20 μm)^26^,^27^,^28^,^29^,^30^,^31^ or with micro/nanofluidic channel constriction^32^,^33^,^34^,^35^,^36^,^37^,^38^. Low-voltage pulses, varying from several to several tens of volt, were found sufficient to concentrate the imposed electric field strength high enough (e.g. 500–1000 V/cm) for successful cell membrane breakdown. These microelectroporation systems open new routes towards the elimination of aforementioned electroporation induced apoptosis and simultaneously offer some other advantages over the commercial systems, namely *in situ* monitoring of intracellular content transport and electroporation dynamics at single cell level^39^,^40^,^41^,^42^,^43^, better accuracy, and flexibility on treatment for different cell populations^44^,^45^,^46^,^47^,^48^,^49^,^50^,^51^,^52^,^53^. However, most of these microelectroporation systems still ignore the variations among individual cells of a large population, leaving many factors still uncontrolled just like in those commercial systems. For example, according to equation 1, the needed transmembrane potential is not only related to the field strength, but also the size and electrical properties of the treated cells. Unfortunately, this issue did not attract enough attentions in the past due to the lack of simple but effective tools. We here propose a Micropillar Array Electroporation (MAE) approach to accomplish size specific electroporation to cells. In MAE, cells are sandwiched between a plain plate electrode and a plate electrode with well-patterned micropillars array on its surface. In this way, the number of micropillars each cell faces varies with its membrane surface area, or the size of cells, as schematically shown in Fig. 1. In another word, large cells receive more electroporation locations and area, which means more transient pores of uniform size are likely created on their cell membrane than that on small cells. In addition, such size dependent pore formation mechanism is not affected by the random dispersion fact of cells as micropillars are well-patterned in a large array configuration. Unlike some pioneer work in which a few micro- or nanoscale pillar electrodes were used as the replacement of capillary electrodes to monitor the intracellular electrical signals of single or a few cells for electrophysiology study (i.e., SCE)^54^,^55^, this new MAE setup utilizes well-patterned, large-scale (centimeter size) micropillar array to achieve size specific treatment to cells of a large population for efficient uptake of exogenous payload (like BE). In fact, it works like many SCE units are carried out in parallel with no need for cell positioning. As every cell electroporation becomes representative, the cellular uptake dynamics study on individual cells in MAE could provide useful information in electroporation protocol identification for unknown cell sources. Therefore, it has potentials to facilitate the communication between SCE (for cell electrophysiology study) and BE (for large scale gene transfection tests) to leverage current electroporation-based delivery technology. In this contribution, we evaluated its transfection enhancement of reporter genes (pMaxGFP and gWizLuc) and their corresponding siRNAs. Several adherent and suspension cell lines including some hard-to-transfect cells and stem cells were tested to demonstrate its broad effectiveness.

## Results

### Enhancement of micropillar array electroporation on reporter gene transfection

We first did MAE electroporation on NIH 3T3 cells and K562 cells for DNA plasmid delivery to demonstrate its effectiveness for both adherent and suspension mammalian cells. For comparison purpose, electroporation using both a commercial system (denoted as “BTX) and another configuration with two closely placed plain electrodes but no micropillar pattern (denoted as “Au Plain Plate”) was also carried out in parallel. Successful transfection is observed in all three cases with many cells expressed green fluorescence protein (GFP) (Fig. 2a and Supp Figure S1). Their quantitative difference on GFP-positive cells was further measured with flow cytometer. As shown in Fig. 2b, the transfection efficiency with two closely placed plain plate electrodes (43.6 ± 1.6% for K562 cells and 44.1 ± 1.8% for 3T3 cells) is generally much better than that from BTX (K562: 25.7 ± 1.8%, 3T3: 25.4 ± 3.6%). When micropillar array is introduced on the electrode surface, the transfection percentage gets further improved (K562: 70.3 ± 2.5%, 3T3: 65.1 ± 3.7%). These results confirm the enhancement of MAE on plasmid transfection to mammalian cells and the improvement is indeed attributed to both the micropillar features and the closely placed electrode configuration. Some loss on the cell viability (~10–15%) is observed, but not statistically significant (Fig. 2c). Such minor viability loss might be the consequence of exposing more cells to those microelectrodes. Although the high-voltage induced apoptosis issues aforementioned are largely mitigated in MAE with its much lower pulse voltage (10 V), the electrochemical hydrolysis of water is not completely eliminated. As the cell suspension was squeezed in the narrow gap between electrodes in MAE, more cells were brought close to the micropillar electrode with larger exposed cell membrane surface. The hydrolysis-associated negative impact on these cells could cause extra loss on the overall cell survival rate. Nevertheless, the cell viability in all these cases is above 70%, acceptable in most biological and therapeutic application requirements.

Despite of the slight sacrifice of cell viability, substantial improvement on DNA transfection is achieved with MAE, as confirmed by Fig. 2b. More cell lines were tested to demonstrate the broad effectiveness of MAE in mammalian cell transfection. As shown in Fig. 3, similar high transfection rates are observed in both common cell lines widely used in transfection tests (>70%) and hard-to-transfect HL-60 cells (~45%) or stem cells (~50%). The cell viability varies with different cell types, ranging from 65 to 95%. It is worth to point out that all these transfection tests were done under the same electric pulse conditions (625 V/cm, single pulse with a duration of 10 ms), which might not be best for some cell types. Nevertheless, it verifies the excellent compliance of MAE in different cell sources while further fine tuning of the pulse conditions may be necessary for the best results.

### Enhancement of micropillar array electroporation on siRNA delivery

To further demonstrate the effectiveness of MAE to RNA interference applications, we chose small interfering RNA (siRNA) with sequences that could specifically silence the expression of GFP or Luciferase. Their knockdown efficiency was evaluated by co-transfecting with pGFP or pLuc plasmids. Considering the importance of RNA interference in cancer treatment, K562 and A549 cells, two model cancer cell lines, K562 for suspension cells and A549 for adherent cells, are used here to examine the effectiveness of siRNA delivery by MAE, respectively. As shown in Fig. 4a, clear suppression of GFP expression is observed when co-delivering pMaxGFP and their corresponding siRNA of 5 pmol to K562 cells in both commercial electroporation system (“BTX”) and MAE. More GFP expression is turned off by MAE, with ~54% and ~18% further drop of GFP level than that in BTX and Au plain plate setup, respectively. The overall knockdown level falls below 30% (Fig. 4b). Down regulation of Luciferase plasmid (“pLuc”) was also evaluated by co-transfecting with its corresponding siRNA (“GL3”). Compared to the knockdown result from BTX, additional ~11% drop of Luciferase signal is found in MAE with 5 pmol siRNA (Fig. 4c). But unlike the knockdown of pMaxGFP, a larger dosage (i.e., 30 pmol) of siRNA GL3 is needed to shutoff the Luciferase expression level below 30% with MAE (Fig. 4c). Similar knockdown enhancement was also observed in A549 cells (Fig. 4b,c), confirming its effectiveness to adherent cells as well. It is worth to point out that as co-transfection of plasmids and siRNA is used here, the delivery enhancement on the targeting reporter gene and its corresponding siRNA occur simultaneously in MAE. It must shut off more proteins than the BTX system to reach the similar protein expression level. Although siRNA probes are introduced into the treated cells by MAE and more corresponding protein expression is effectively suppressed, only minor cell viability loss is found for both cell types (Fig. 4d and Supp Figure S2).

### Effect of size and density of micropillars on electroporation enhancement

The dimensions of individual micropillar and their pitch size in the array affects the enhancement of cell transfection in MAE as they determine the number of micropillars each cell faces during electroporation. To simplify the case, we fabricated micropillars of two different sizes (6 μm and 2 μm in diameter with the same pitch size of 2 μm). As shown in Fig. 5a, K562 cells face ~2–4 6-μm micropillars on average (full coverage) with a maximum of 6–9 micropillars (partial coverage) for some giant cells. Many covering events are incomplete and heterogeneous, largely depending on the actual size of cells and their settling locations on micropillars. As comparison, on a 2-μm micropillar electrode, more micropillars cover each cell (with as many as 16 for some large cells) and incomplete coverage is hardly observed despite the random location of cells (Fig. 5b). Therefore, the cell coverage on 2-μm micropillars varies more accurately with the actual size of individual cells. As the consequence, the number of locally porated openings and the total effective permeable area on the cell membrane should become more size specific and the DNA delivery dosage to cells of various size populations get improved. As demonstrated in Fig. 5c, the transfection efficiency of 2-μm micropillar MAE is ~65%, ~70%, and 71% for 3T3 cells, K562, and A549 cells, respectively, while only ~55%, ~59%, and ~61% for those using 6-μm micropillars, though both were much higher than the one using Au parallel plain plate electrodes separated by the same distance or bulk electroporation using cuvettes. This additional gain on the transfection efficiency is believed the result of more accurate and uniform allocation of pulse on cells based on their size in 2-μm micropillar MAE. Its electroporation works more effectively to cells of different sizes than the 6-μm ones and accomplishes better transfection performance.

## Discussions

Such transfection enhancement of MAE is attributed to the synergistic effects of the electric field focusing, localized electroporation, and size-dependent treatment. The first two effects benefit for cell membrane permeabilization at benign pulse conditions and its better recovery afterwards, while the size-dependent treatment allocates the number and area of the transient openings on individual cell membrane to ensure homogeneous treatment on cells of various sizes. Their specific contributions are addressed as following:

### Focusing the electric pulses locally

Like what occurs in many micro/nanofluidic electroporation proof-of-concepts^24^,^25^,^26^,^27^,^28^,^29^,^30^,^31^,^32^,^33^,^34^,^35^,^36^,^37^,^38^,^39^,^40^,^41^,^42^, micropillars in MAE help focus the electric field with their microscale far end that protrudes towards the cell membrane (Fig. 6a,b). According to the continuity of the electric field, the focusing level depends on the surface area (or size) of micropillars. As the focused electric pulses affect mainly a tiny portion of the cell membrane each micropillar faces, this gives additional localized electroporation benefit on the subjected cell. But unlike micro/nanofluidic electroporation, MAE does not require fluidic components to trap cells to accomplish these benefits. Its operation is therefore more compatible and similar to the commercial electroporation systems. As demonstrated by the COMSOL simulation (Fig. 6c), the transmembrane potential near micropillar on a suspended spherical cell is much higher than that without micropillar when the overall field strength is held constantly at 625 V/cm for both cases. As a consequence, these locations more incline to form temporary openings than elsewhere during electroporation.

Physical observation of such millisecond membrane opening process in live cell electroporation is still very challenging, considering the requirement on integrating the electroporation setup within Cryo-TEM facility^56^. However, reasonable speculation based on current available electroporastion theory and some recent cell electroporation simulation findings^57^,^58^ could approximately reveal the pore formation dynamics occurs in MAE. According to equation (1), transmembrane potential on an individual cell also varies by locations or the local cell surface orientation to the imposed electric field. In bulk electroporation, this means the highest transmembrane potential appears in two locations (i.e., 0 and 180 degrees, facing the two large plate electrodes) of individual cells and drops continuously in between, according to their suspension status and spherical geometry^57^,^58^. As the consequence, heterogeneous permeability presents across the whole cell membrane: with some large pores close to the two poles of the spherical cell and many other small, incomplete openings elsewhere. On the contrary, as the electrical pulses are highly focused by many tiny micropillars of the same size, the local transmembrane potential in locations facing these microelectrodes on an individual cell is similar in MAE. Small pores of similar size are therefore generated in these locations and distributed evenly on the cell membrane. Although the input total energy in both systems (bulk electroporation and MAE) is same, different polarization consequence occurs on individual cells for their different electrode configurations: more small pores of uniform size are generated in MAE while a mixture of large pores and many other small, incomplete openings in bulk electroporation.

### Transfecting cells size dependently

The number of induced transient pores and the overall effective permeable area on the cell membrane varies with the size of individual cells when the design of micropillar array is fixed. With well-patterned array configuration, such size-dependent treatment of MAE is not affected by the randomly located sites of cells. In another word, a big cell faces more micropillars and should have more porated locations to facilitate cellular uptake. To verify our hypothesis, the transgene expression of pGFP inside individual cells was measured, together with their cell size using NIH Image J. According to our size specific electroporation rationale, the size of cells matches to the number of micropillars they face, regardless their random dispersion. Therefore, the cellular uptake of DNA plasmids for cells of different sizes also represents similar relation to the number of micropillars they have faced early. As shown in Fig. 6d, despite of the large scattering of data, the GFP intensity clearly shows a proportional increase with the cell size, particularly for large cells (>10 μm). Different from the BTX system whose GFP signal is accumulated mainly in a specific size range (<12 μm), the signal from MAE is stronger and extends to a broader size range. Similar trends are also observed in the dot-plots of the flow cytometry results (Figures supp S3a and S3b). This suggests that MAE works effectively to cells of many different sizes, unlike the commercial system which works best for cells of certain size populations. This is reasonable as the recommended electroporation protocols for most commercial systems are generally identified by trial-and-error processes and their optimal performance must be tied with effective transfection to cells of the dominated size populations.

To conclude, our MAE system could enhance the electroporation-mediated DNA and RNA delivery to both adherent and suspension cells. Its well-defined micropillar array configuration ensures size specific treatment to a large number of cells regardless their random dispersion. Besides the benefits we demonstrated here, the cellular uptake dynamics of individual cells in MAE could be representative as it works like many size-dependent treatments are done in parallel. This could provide useful information to help simplify the tedious, cell-specific protocol searching process for bulk electroporation and help mutual support between two long separated electroporation fields, single cell electroporation and bulk electroporation. Its success may benefit many research communities where a safe and effective non-viral gene delivery approach is needed on a daily basis.

## Methods

### Materials and reagents

DNA plasmids with gWiz Luciferase and pMax GFP reporter genes were purchased from Aldevron, Inc and Lonza, Inc respectively. Small interfering RNA (siRNA) used for silencing GFP (expressed by pMaxGFP) and Luciferase genes were synthesized by Thermo Scientific (Pittsburgh, PA) and the sequences were as follows: siRNA for GFP silence, sense strand, 5′-CGCAUGACCAACAAGAUGAUU-3′; antisense strand, 5′-UCAUCUUGUUGGUCAUGCGGC-3′; Luciferase GL3 Duplex (Luc-siRNA), sense strand, 5′-CUUACGCUGAGUACUUCGA-3′; antisense strand, 5′-UCGAAGUACUCAGCGUAAG-3′. All other chemicals were purchased from Sigma-Aldrich and the cell culture reagents were purchased from Life Technologies (Carlsbad, CA) unless specified.

### Micropillar array electrode fabrication

**Figure 1:**
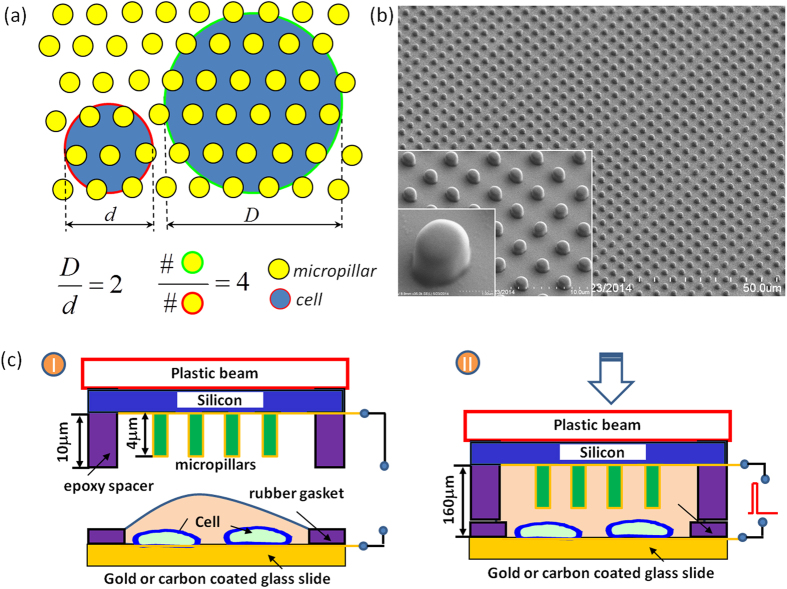
The working principle of the micropillar array electroporation (MAE). (**a**) Schematic of the cell size-specific treatment mechanism (large cells face more micropillars with each providing focused electric pulse during electroporation); (**b**) a SEM image of 2-μm micropillars; (**c**) schematic illustration of MAE operation.

**Figure 2:**
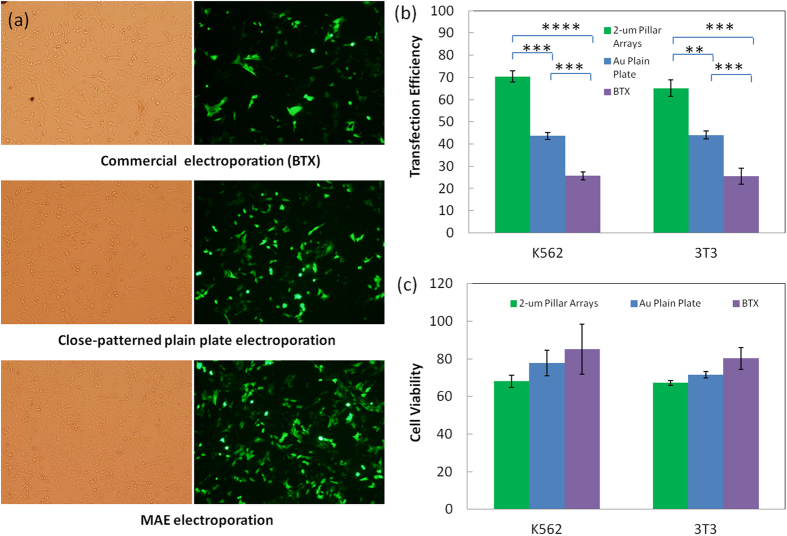
Transfection enhancement of pGFP plasmids in 2-μm micropillar MAE. (**a**) Phase contrast and fluorescence microscopic images of NIH 3T3 cells after transfection by a commercial system (“BTX”), “Au plain plate”, and MAE; quantitative results of transfection efficiency (**b**) and cell viability (**c**) for 3T3 cells and K562 cells, respectively. n = 6 with (**) represents p < 0.01, (***) represents p < 0.005, (****) represents p < 0.0001.

**Figure 3:**
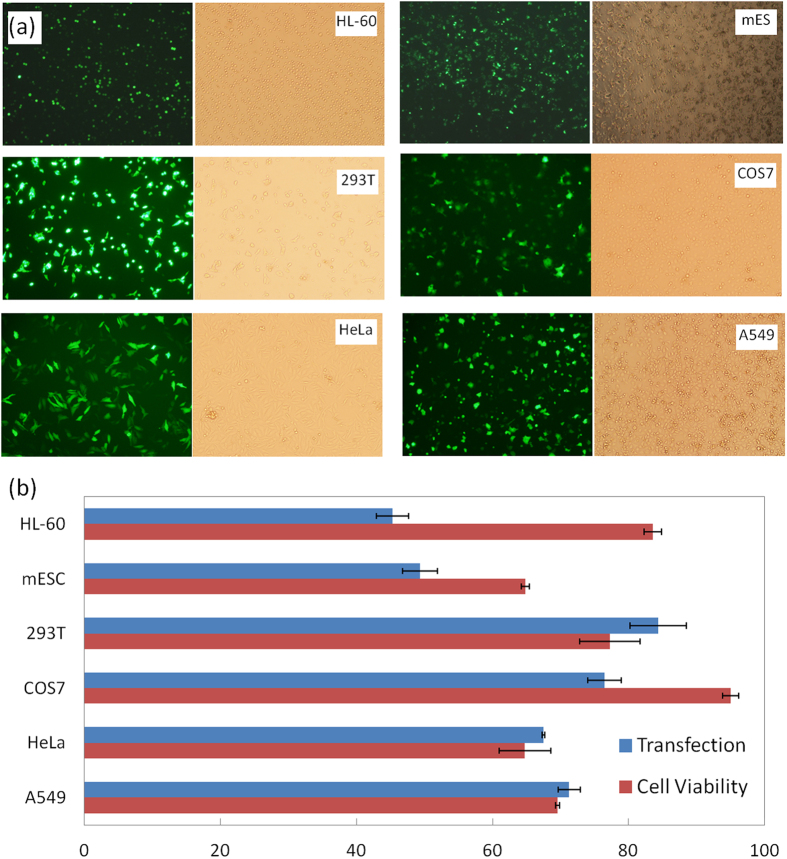
Transfection performance of pGFP plasmids in other mammalian cell lines with 2-μm micropillar MAE.

**Figure 4:**
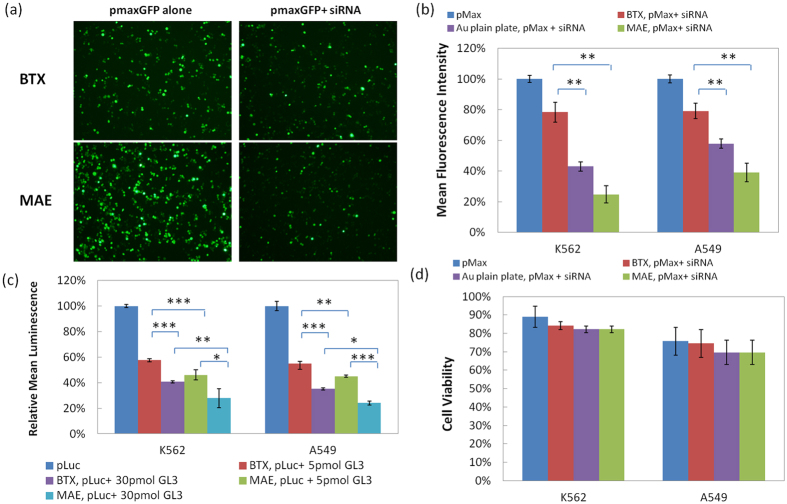
Enhancement on siRNA delivery in 2-μm micropillar MAE. (**a**) Fluorescence images of K562 cells and (**b**) fluorescence intensity measurement on GFP expression level in K562 and A549 cells when co-transfecting pMaxGFP and its corresponding siRNA; (**c**) the luminescence measurement on Luciferase expression level in K562 and A549 cells when co-transfecting pLuc and knockdown siRNA probe (“GL3”); (**d**) cell viability of K562 and A549 cells in panel (**b**). Triple duplicates with (*) represents p < 0.05, (**) represents p < 0.01, (***) represents p < 0.005.

**Figure 5:**
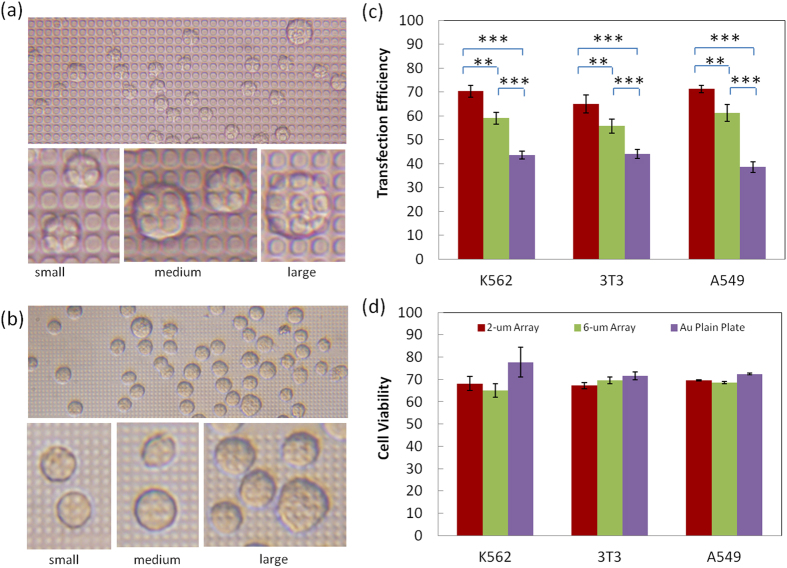
The effect of micropillar size and density on MAE electroporation enhancement. (**a**,**b**) Phase contrast images of cell coverage on 6-μm micropillar array (**a**) and 2-μm micropillar array (**b**); the comparison on the enhancement performance for MAE based on micropillars of various sizes: transfection efficiency (**c**) and cell viability (**d**). Triple duplicates with (**) represents p < 0.01, (***) represents p < 0.005.

**Figure 6:**
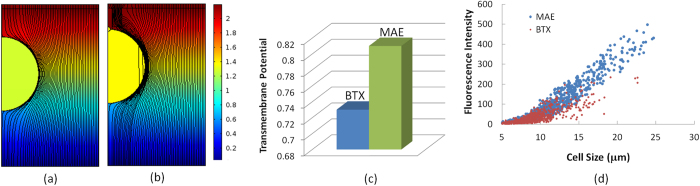
(**a**,**b**) COMSOL simulation of the calculated electric potential and field lines around a cell facing an “Au plain plate” electrode (**a**) and a 2-μm micropillar (**b**,**c**) the calculated transmembrane potential at location “A” (marked in Figure S5) of the cell; (**d**) the plot of fluorescence intensity of GFP in transfected K562 cells to their size in a commercial bulk electroporation system (“BTX”) and a 2-μm micropillar MAE system.

Micropillar arrays were fabricated by BioMEMS technologies. Briefly, SU-8 photoresist was patterned on a Si (100) wafer via photolithography. Micropillars of 2 or 6 μm in diameter and a pitch size of 2 μm (Fig. 1b) were defined in several 12-mm disc regions (Supp Figure S4). The actual height of the finished micropillars was found to be ~4 μm. Conductive micropillars were made by sputter coating with gold. A second SU-8 layer was then applied to cover the non-electrode area and define two 100-μm long, 20-μm wide connecting channels to the micropillar array chamber, one on each side (Supp Figure S4). When a drop of cell solution is squeezed to fill the entire micropillar chamber, the extra solution is guided into these channels and push air out to avoid potential bubble trapping issues during the chamber closure. Ball wire bonding was applied to connect the microelectrodes to wires that were plugged to a pulse generator (BTX 830).

### Cell culture

NIH/3T3 cells (ATCC, CRL-1658) were routinely grown and maintained in high glucose DMEM supplemented with 10% newborn calf serum (NCS), 1% penicillin and streptomycin, 1% L-glutamine and 1% sodium pyruvate. K562 cells (ATCC, CCL-243), A549 (ATCC, CCL-185), HeLa (ATCC, CCL-2), COS-7 (ATCC, CRL-1651), 293T (ATCC, CRL-3216), and HL-60 (ATCC, CCL-240) were cultured in RPMI 1640 supplemented with 10% NCS, 100 U/mL penicillin, 100 μg/mL streptomycin, and 100 μg/mL L-glutamine. Mouse embryonic stem (ATCC, CRL-1934) were cultured on gelatin-coated tissue culture flasks and maintained in an undifferentiated state using Dulbecco’s Modified Eagle’s Medium (DMEM with 4.5 g/l D-glucose) supplemented with 15%(v/v) fetal bovine serum (FBS), 100 U/ml penicillin, 100 μg/ml streptomycin, 0.1 mM non-essential amino acids, 10 ng/ml murine recombinant leukemia inhibitory factor (LIF), 0.1 mM monothioglycerol, 2 mM L-glutamine (Sigma Aldrich, St. Louis, MO) and 1 mM sodium pyruvate (Invitrogen). All cultures were maintained at 37 °C with 5% CO_2_ and 100% relative humidity.

### Electroporation setup and process

Cells were first centrifuged and re-suspended in fresh OPTI-MEM I (a serum free medium) at a density of 0.5 × 10^6^ cells/mL. Plasmid DNA (pGFP or pLuc) of 10 μg was then added to make the electroporation sample solution. When co-tranfecting DNA plasmid and siRNA, additional 5 pM or 30 pM siRNA probes were added in medium with plasmid DNA by gentle mixing with pipette. Immediately following the addition of DNA and/or siRNA probes, the mixture was loaded into a cuvette (BTX) or the chamber of MAE for transfection.

In MAE electroporation (Fig. 1c), a piece of gold coated plate electrode with a PDMS gasket of 200 μm in height was first mounted on a mini mechanic press. One drop of cell solution (20 μL) was then loaded into the created liquid holding chamber. The micropillar array electrode, mounted on the other plate of the press, was loaded down to squeeze the liquid drop until the edge of the pre-defined SU-8 spacer (~10 μm) surrounding micropillars firmly touched the PDMS gasket to seal the liquid chamber. The SU-8 spacer protects micropillars from destruction and controls the gap between the two electrodes of MAE. A single, 10-ms electric pulse of 10 V was then applied across the two electrodes for electroporation. For comparison, standard electroporation was also done using a commercial BTX system (ECM 830, Harvard Apparatus). Samples of 100 μL each were loaded into electroporation cuvettes with the parallel electrodes separated by 2 mm and a standard electroporation protocol (125 V, single 10-ms pulse) was applied. As the measured gap size between the two electrodes of MAE system is ~160 μm (see supplemental materials for details), this is designated to ensure that the overall electric field strengths are the same (625 V/cm) in all three systems. After treatment, cells were transferred to 6-well plates and cultured for another 24 hr and then harvested for analysis.

### Transfection efficiency and cell viability

The expression of pGFP plasmids was evaluated both qualitatively by visualizing cells with green fluorescence within some representative areas under an inverted fluorescence microscope (Olympus, Japan) and quantitatively by counting cells using an Agilent 2100 Bioanalyzer (Agilent Technologies, Santa Clara, CA). The fluorescence intensity of GFP was measured using the Cell Assay Module with live cells stained with carboxy-naphthofluorescein (CBNF). The results were analyzed with Agilent 2100 Expert Software and 500–1,500 events were counted for each sample. The transfection efficiency of pGFP is defined as the number of cells emitting fluorescence signal to the total number of cells in a sample (gated fluorescence signal of GFP). The Luciferase expression was quantified by One-Glo^TM^ Luciferase assay system (Promega, Madison, WI). One-Glo^TM^ reagent of 100 μL was added to the cell growth medium of 100 μL in 96-well plate. Luminescence was measured with a plate reader (FLUOstar OPTIMA, BMG LABTECH, Germany) after 10 min incubation at room temperature for complete cell lysis. The transfection efficiency of pLuc is presented as the luminescence of the total live cells in a sample. The down regulation efficiency of siRNA is normalized to the expression of the corresponding gene (pGFP or pLuc) when they are delivered alone.

The cell viability was evaluated by an MTS cell proliferation assay (Promega, Madison, WI). Briefly, 100 μL cells from each sample 24 hr post electroporation were transferred to a 96-well plate and CellTiter 96 AQueous One solution (Promega, Madison, WI) of 20 μL was added to each well and all samples were incubated at 37 °C for another 4 hr. Absorbance was measured at 492 nm on an automated plate reader (Elx 800, Biotek, VT). Data points were represented as the mean ± standard deviation (SD) of triplicates, unless otherwise indicated. The cell viability is calculated as the absorbance signal ratio of an electroporated cell sample to that of the negative control cell sample in MTS assay, after extracting the absorbance background from the media.

### Simulation on the electric field of MAE

COMSOL (Mathworks, MA), was used to calculate the electric field in MAE based on a finite-element method (FEM). We considered an axial symmetric model with one micropillar (2 or 6 μm in diameter) and a single cell (d = 16 μm) in the computation domain (35 μm × 21 μm). An electric field (E = 625 V/cm) was assigned across the top and bottom of the computation domain whose right side boundary was set as insulated wall. The cell was placed at the center of the left side boundary (the symmetrical axis) and a three-layer cell model, divided as the external medium, the cell membrane (5 nm in thickness), and the cell cytoplasm, was setup^57^,^58^. A gold micropillar was placed at the top of the cell, 0.5 μm and 1.0 μm away from the cell and the symmetrical axis, respectively. With a pitch size of 2 μm, cell membrane deformation in the gap of micropillars seems essential^59^. Therefore, a quarter-circle raised arch (with a radius of 0.5 μm) was created on the cell membrane close to the micropillar to mimic its deformation. Detailed model dimensions and mesh setup are illustrated in Supp Figure S5. The electric potential distribution around the micropillar and the cell was calculated. In this three-layer cell model, the electrical conductivity of buffer, cytoplasm, membrane, and gold-coated micropillar was set as 0.8, 0.2, 5 × 10^−7^, and 4 × 10^7^ S/m, respectively.

### Statistic Analysis

All significance analysis was done on triple duplicates unless specified, with two-tailed *t*-test.

## Additional Information

**How to cite this article**: Zu, Y. *et al*. Size Specific Transfection to Mammalian Cells by Micropillar Array Electroporation. *Sci. Rep.* **6**, 38661; doi: 10.1038/srep38661 (2016).

**Publisher's note:** Springer Nature remains neutral with regard to jurisdictional claims in published maps and institutional affiliations.

## Supplementary Material

Supplemental Materials
