# Supplementary material for: Size Specific Transfection to Mammalian Cells by Micropillar Array Electroporation
Source: Sci Rep. 2016 Dec 7;6:38661. doi: 10.1038/srep38661 (PMC5141490; doi:10.1038/srep38661)
Supplement: Supplemental Materials [file srep38661-s1.pdf]

Supplemental Materials for

Size Specific Transfection to Mammalian Cells by Micropillar Array Electroporation

Yingbo Zu[a,b], *Shuyan Huang*[c,d], *Yang Lu*[a,b], *Xuan Liu*[c,d], and *Shengnian Wang*[a, b, d*]

[a] *Chemical Engineering,* [b] *Institute for Micromanufacturing,* [c] *Biomedical Engineering,*

[d]*Center for Biomedical Engineering and Rehabilitations, Louisiana Tech University, PO Box 10137, Ruston, LA, 71272 USA*

[*]Corresponding author:

Tel: +1 (318)257-5125; Fax: +1 (318) 257-5104; E-mail: swang@latech.edu

Supplemental Figures

Figure S1. Fluorescence and phase contrast microscopic images of pGFP plasmid transfection by a commercial system (“BTX”) and MAE on K562 cells.

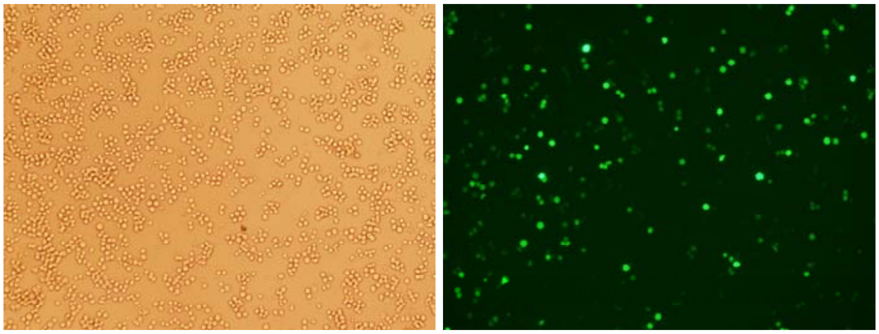

Commercial electroporation (BTX)

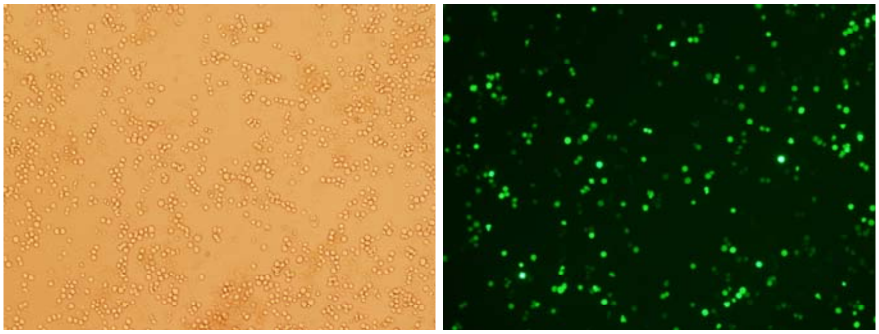

Close-patterned plain plate electroporation

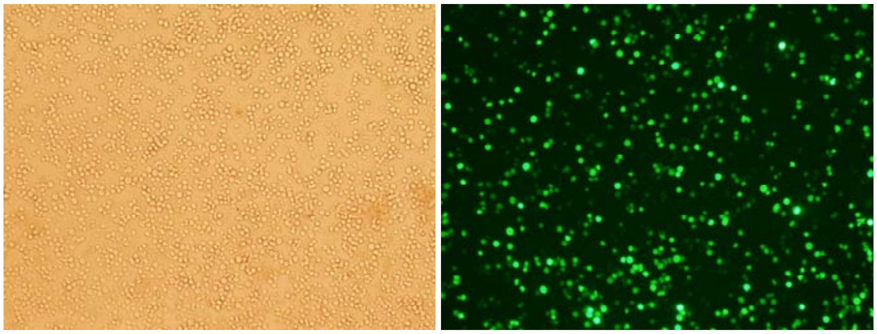

MAE electroporation

Figure S2. Cell viability of K562 and A549 cells for cotransfection of pLuc and siRNA GL3 in panel c of Figure 4.

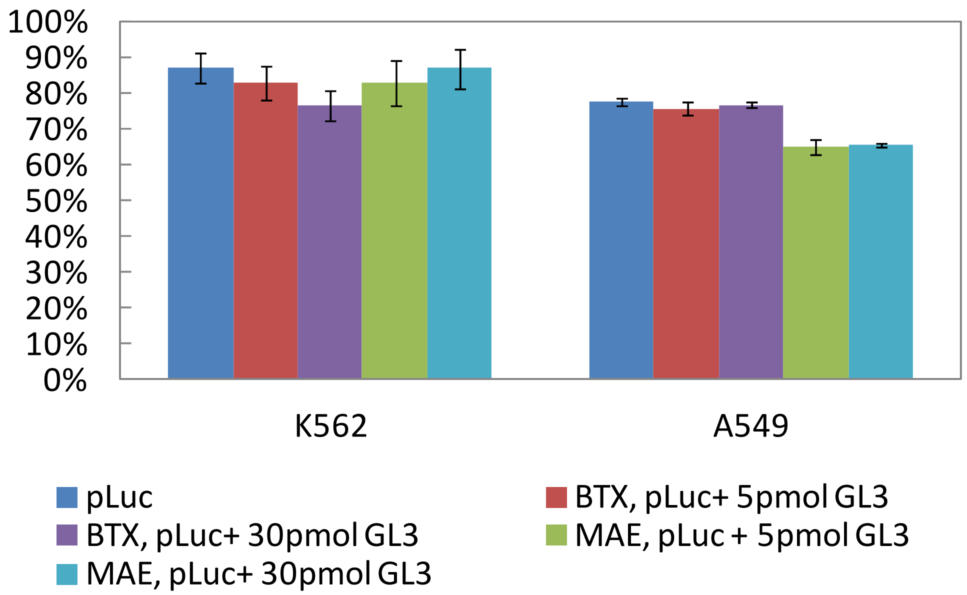

Figure S3. The dot-plots of fluorescence intensity of GFP in transfected K562 cells to the cell size in a commercial bulk electroporation system and a 2-μm micropillar MAE.

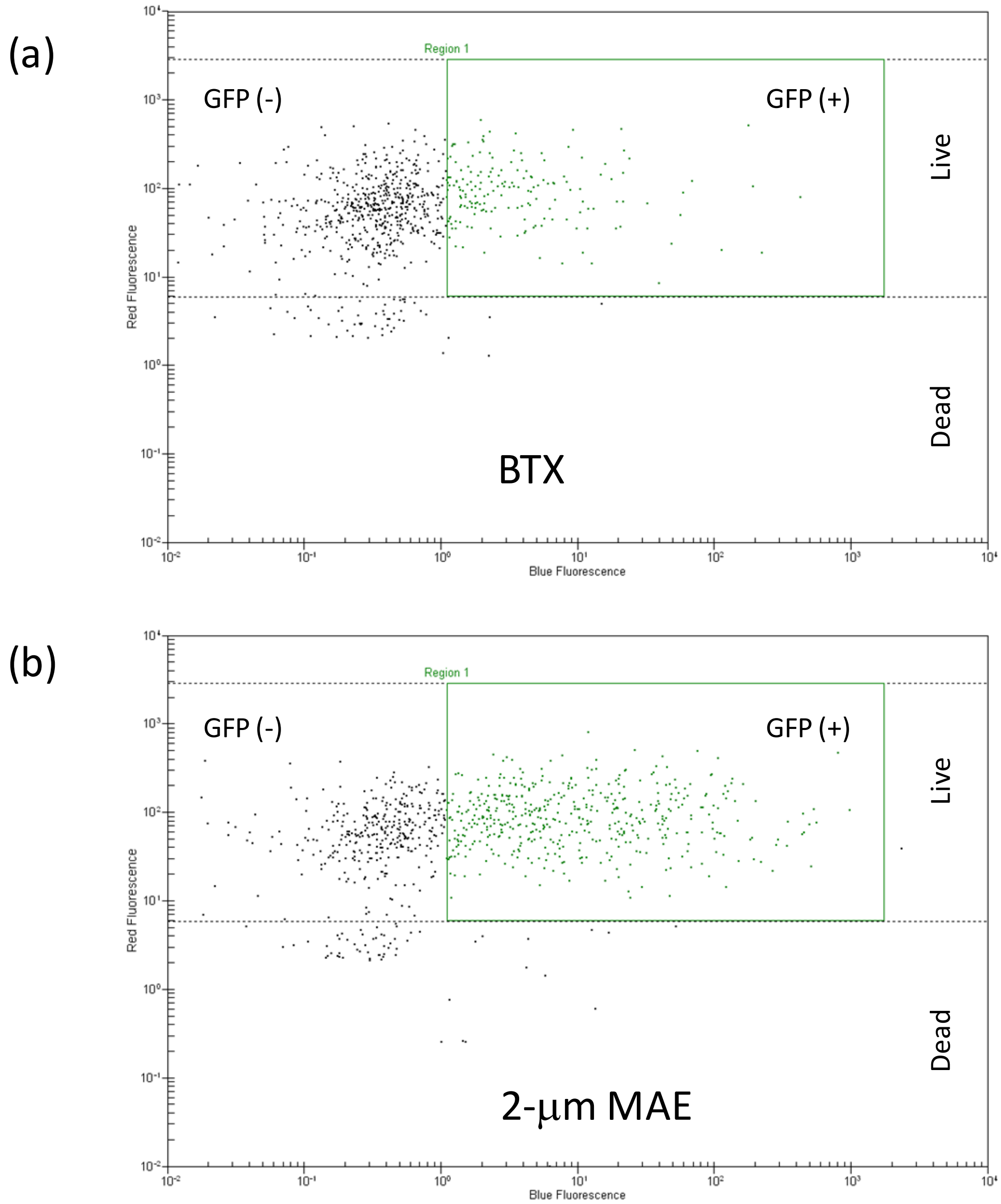

Figure S4. Schematic of the micropillar array electrodes integrated with the SU-8 spacer and connecting microchannel in MAE.

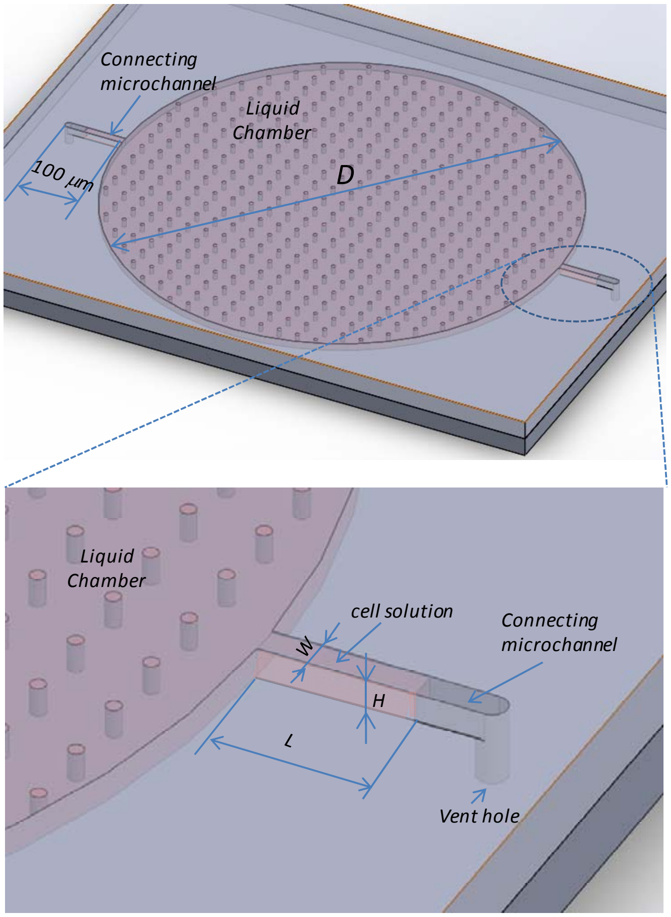

Figure S5. The model geometry and mesh setup for the electric field simulation of a single micropillar protruding towards a single cell. Location “A” marked is the chosen point in later transmembrane potential calculation.

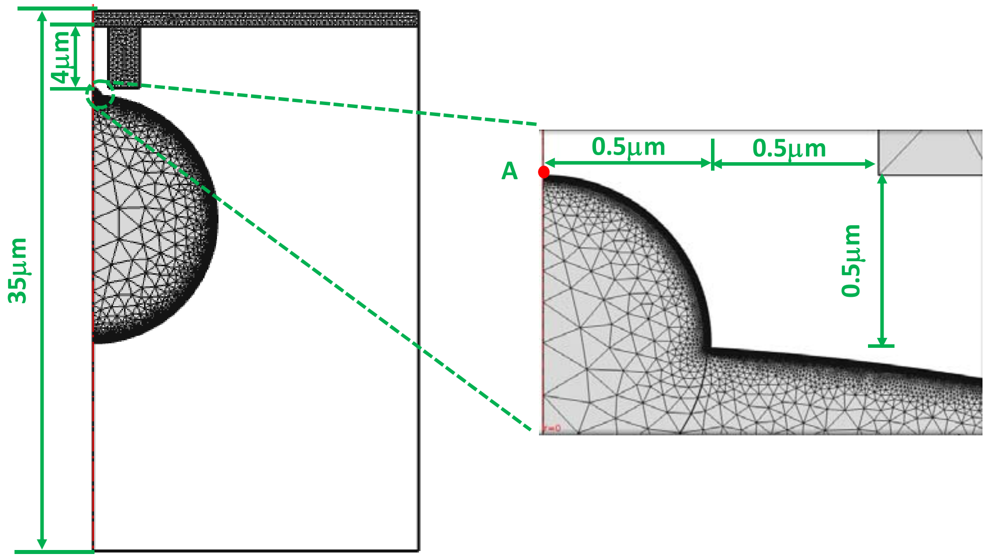

Supplemental Text

Measurement of the Gap Size of Electrodes in MAE

As the sealing of the liquid chamber between the two electrodes in the MAE system and "Au plain plate" systems was done by a PDMS gasket, some deformation occurred when the top micropillar electrode was firmly pressed down for closure. Therefore, the actual gap size between the two electrodes was smaller than the sum of the measured thickness of the epoxy spacer (10 μm) and the PDMS gasket (200 μm). To find out the actual distance between the two electrodes, we measured the amount of excess liquid in the connecting channels that was squeezed out of the liquid chamber when the two plates were closed (see Figure S4). The actual gap size was then calculated based on the total volume of the loaded cell solution and the dimensions of the liquid chamber and the connecting channels.

$$V_{\text{total cell solution}} = V_{\textit{cell solution in liquid chamber}} + V_{\text{cell solution in connecting channel}} \\ = \pi \mathrm{D}^2 H / 4 + 2WHL = (\pi \mathrm{D}^2 / 4 + 2WL) H$$

where *D* is the diameter of the liquid chamber, *W* is the width of the connecting channel, *L* is the length of liquid the connecting channel and *H* is the height of the gap between the two electrodes (see Figure S4). The gap size, H, is then calculated by

$$H = \frac{V_{\text{total cell solution}}}{\pi \mathrm{D}^2 / 4 + 2WL}$$
